# Thermal and Rheological Properties of a Family of Botryosphaerans Produced by *Botryosphaeria rhodina* MAMB-05

**DOI:** 10.3390/molecules16097488

**Published:** 2011-09-02

**Authors:** Paulo R.M.S. Fonseca, Robert F.H. Dekker, Aneli M. Barbosa, Joana L.M. Silveira, Ana F.D. Vasconcelos, Nilson K. Monteiro, Gabriel Aranda-Selverio, Maria de Lourdes Corradi da Silva

**Affiliations:** 1 Department of Physics, Chemistry and Biology, Faculty of Science and Technology, University Estadual Paulista, Presidente Prudente 19060900, Brazil; Email: prmsf@yahoo.com.br (P.R.M.S.F.); anaflora@fct.unesp.br (A.F.D.V.); monteironk@yahoo.com.br (N.K.M.); gabrielselverio@yahoo.com.br (G.A.S.); 2 Biorefining Research Initiative, Lakehead University, Thunder Bay, ON, P7B 5E1, Canada; Email: rdekker@lakeheadu.ca; 3 Department of Biochemistry and Biotechnology, CCE, State University of Londrina, Londrina 86051990, Brazil; Email: anelibarbosa@gmail.com; 4 Department of Biochemistry and Molecular Biology, Federal University of Paraná, Curitiba 81531990, Brazil; Email: jlms12@yahoo.com

**Keywords:** β-glucans, botryosphaeran, thermal analysis, FT-IR spectroscopy, rheology

## Abstract

Differential scanning calorimetry (DSC), thermogravimetry (TG) and Fourier-transform infra-red spectroscopy (FT-IR) analyses were performed to investigate changes in the physico-chemical properties of botryosphaerans, a family of exopolysaccharides (EPS) produced by the fungus *Botryosphaeria rhodina* MAMB-05 grown on glucose (EPS_GLC_), sucrose (EPS_SUC_) and fructose (EPS_FRU_). A slight endothermic transition and small mass loss attributable to the removal of water of hydration were observed in the DSC and TG analyses, respectively, for the three EPS samples. The FT-IR spectra confirmed no structural changes occurred during thermal treatment. Viscometry was utilized to obtain information on the rheological behaviour of the EPS in aqueous solutions. The Power Law and Cross Equations determined the natural pseudoplastic characteristics of the EPS. Comparatively, results obtained for EPS produced when *B. rhodina* MAMB-05 was grown on each of the three carbohydrate sources demonstrated similar apparent viscosity values for EPS_GLC_ and EPS_SUC_, while EPS_FRU_ displayed the lowest apparent viscosity of the three botryosphaerans, suggesting a higher degree of ramification and lower Mw. EPS_GLC_ and EPS_SUC_ possessed similar degrees of ramification. The slight differences found in their viscosities can be explained by the differences in the type of branching among the three botryosphaerans, thus varying the strength of intermolecular interactions and consequently, consistency and viscosity. The physico-chemical studies of botryosphaerans represent the originality of this work, and the knowledge of these properties is an important criterion for potential applications.

## 1. Introduction

Biopolymers such as the polysaccharides find wide commercial applications because of their capacity to form viscous solutions or dispersions at low concentrations when hydrated [1,2]. The properties of some of these polysaccharides, such as thickening or gelling effects, the ability to stabilise emulsions or suspend solid particles, and the control of stability and texture of some products, find use mainly in the pharmaceutical and food industries [3,4]. Because biopolymers are abundant and largely derived from renewable resources, they are relatively inexpensive, non-toxic and amenable to both chemical and biochemical modifications tailored for specific applications [2]. Furthermore, the stabilising properties of these polysaccharides have also found widespread use in the preservation of fragile biological materials [5]. There is increasing interest in exopolysaccharides (EPS) derived from microbial species as potential substitutes for plant and algal-derived gums. 

An important criterion in considering potential applications of EPS is that knowledge be available about their physicochemical properties ‑ thermal stability, rheology ‑ as well as possible molecular structural modifications that may occur during their handling and treatment. For example, their use in medical therapy requires that the EPS be sterilised, and this is generally achievable through autoclaving. It is therefore important that following thermal treatment, the EPS maintains its original conformation in order not to lose any of its desirable physical, chemical and biological properties.

Thermal analysis studies, such as differential scanning calorimetry (DSC) and thermogravimetry (TG), are common on polysaccharides [1,6,7] because they determine the effects of temperature associated with physical and/or chemical alterations, such as phase transitions and dehydration reactions (endothermic effects), and crystallization and oxidation (exothermic effects) [8]. Infra-red spectroscopy (FT-IR) is frequently used with polysaccharides [9], as this largely determines their structural features such as macromolecular conformation, and inter- and intramolecular interactions [10].

The group of EPS known as the β-glucans display high viscosity in aqueous solutions due to their high molecular weight (Mw), conformation and interactive properties. Since potential use of β-glucans as food hydrocolloids has been proposed based upon their rheological characteristics [11], it is necessary to have knowledge of these properties. Some parameters important in comparing EPS are, for example, consistency index (k, related to molecule relaxation time) and zero-shear rate viscosity (η_0_) [7,12]. Fluids that present a linear relationship between shear rate (γ) and shear stress (τ) are called Newtonian, and when this relationship is not maintained, they are considered non-Newtonian. Aqueous solutions of polysaccharides in may behave as non-Newtonian or Newtonian fluids. The viscosity is a function of shear rate and depends on the molecular weights and the polymer concentration [13]. The most important of these are the pseudoplastic group, where shear rate is dependent only upon shear stress and the apparent viscosity, which decreases as shear rate increases [14].

The fungus *Botryosphaeria rhodina* MAMB-05 produces an exopolysaccharide responsible for the high viscosity of the culture medium when cultured on glucose as sole carbon source [15]. This EPS, named botryosphaeran, forms a strong gel, and was characterized as a (1→3; 1→6)-β-D-glucan [16]. Furthermore, a family of botryosphaerans were produced when *B. rhodina* MAMB-05 was cultured on sucrose and fructose as carbon sources [17].

The triple helical conformation of polysaccharide structure in non-cellulosic β-glucans, which is stabilized by hydrogen bonds between the hydroxyl groups of the polysaccharide chains, appears to be responsible for the stable behaviour of these macromolecules. The presence of (1→6)-β linked gluco-oligosaccharide side-branches does not appear to interfere with the formation of the triple helix [18,19].

The aim of the present study was to investigate the physicochemical properties, thermal and rheological behaviour of botryosphaerans utilizing thermal, FT-IR and viscometry techniques. The botryosphaerans utilized in this paper were produced by *B. rhodina *MAMB-05 grown on three different carbohydrate substrates as sole carbon sources: glucose, sucrose and fructose, and are designated EPS_GLC_, EPS_SUC_ and EPS_FRU_, respectively.

## 2. Results and Discussion

### 2.1. DSC Studies

**Figure 1:**
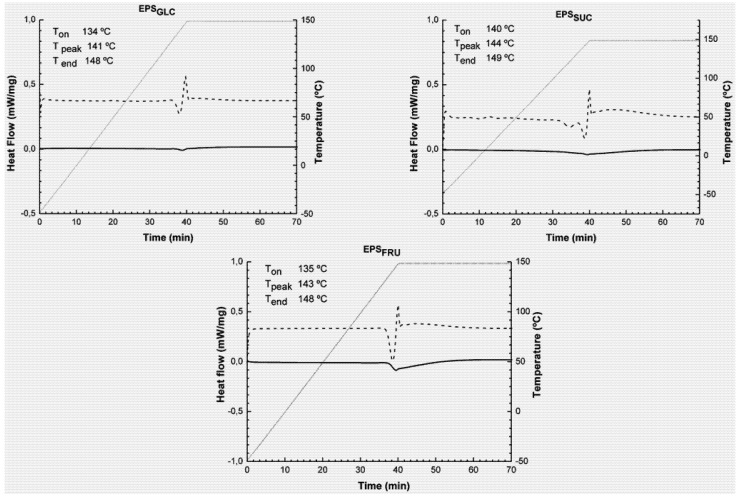
DSC (—) and DDSC (---) curves for botryosphaerans EPS_GLC_, EPS_SUC_ and EPS_FRU_ over the 50–150 °C temperature range. The heating rate was 5 °C/min, with an isothermal hold at 150 °C for 30 min.

Figure 1 shows that the EPS material behaved in a stable manner and did not present either exo- or endothermic peaks that are characteristic of physical and chemical transformations of the EPS. Only a small endothermic variation was observed, beginning at 137 °C, and more pronounced for EPS_FRU_. This slight transition is more visible on examining the derivative curve (Figure 1), and presented evidence of the start (T_on_), middle (T_peak_) and end (T_end_) temperatures of the DSC process. The slightly higher temperature of EPS_SUC_ in relation to EPS_GLC_ and EPS_FRU_ can be due to some conformational variability of the molecules, since these EPS are derived from different carbon sources. However, the stable behaviour did not change with structural differences between the EPS molecules.

An endothermic transition was observed within a 134–149 °C temperature range, which is considered to be relatively high and can be attributed to water removal through evaporation since EPS is a very hydrophilic molecule. In a thermal study on natural and modified gums, Zohuriaan *et al. * [2] found endothermic peaks in the 63–143 °C temperature range, which they attributed to loss of water. Generally, depolymerisation and pyrolytic decomposition of polysaccharides occurs at much higher temperatures: about 350 °C [2]. To confirm that the observed endothermic variations corresponded to water of hydration, DSC was performed on EPS_GLC_, EPS_SUC_ and EPS_FRU_ beginning at ambient temperature and increasing to 200 °C (heating rate of 5 °C/min). Thereafter, the material was immediately cooled to 30 °C followed again by heating to 200 °C at the same heating rate as before. Results obtained for EPS_GLC_, EPS_SUC_ and EPS_FRU_ were similar: in the first heating run there was an endothermic variation at about 145 °C, which did not occur during the second run. A likely explanation for this observation is that the EPS was freed from hydration water after the first heating step, and endothermic variation corresponded to loss of these water molecules, which did not appear during the second heating run. This observation indicated that there was no change in the chemistry of the main polymer chain.

### 2.2. Thermogravimetric Studies

**Figure 2:**
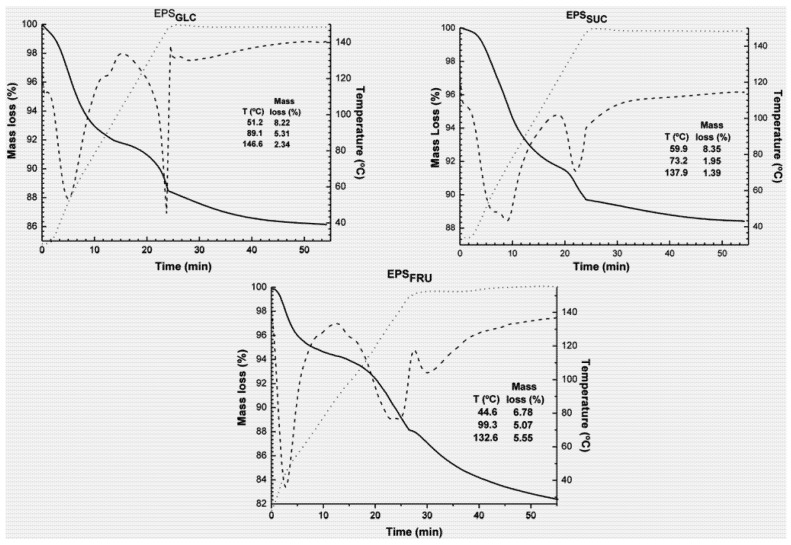
TG (—) and DTG (---) curves for botryosphaerans EPS_GLC_, EPS_SUC_ and EPS_FRU_ within the 30–150 °C temperature range. The heating rate was 5 °C/min, with an isothermal hold at 150 °C for 30 min.

Thermogravimetric (TG) analysis was employed to study the thermal stability of EPS. This determined the mass loss (%) during the heating interval of the sample. Figure 2 shows the TG curve profiles of the EPS samples, demonstrating the similar thermal behaviour of all three botryosphaerans. The mass loss of EPS can be divided into three stages, although it refers to removal of water molecules (*i.e.*, water of hydration). The initial mass loss demonstrated by the first peak on the derivative curve (DTG), refers to water absorbed by the material during preparation for analysis, because the heating stage was conducted in an open pan. The second large peak, at 132–146 °C, was related to a small endothermic transition found by DSC analysis, and was also a consequence of removal of hydration water. The mass loss by EPS_SUC_ appeared to start at a slightly higher temperature in relation to EPS_GLC_ and corresponded to what was found by DSC analysis. The third and last stage corresponded to a mass loss by evaporation, and remained virtually constant as this occurred where there was no further rise in temperature. Figure 2 shows the mass losses in terms of percentages, and was highest for EPS_FRU_ (17.40%), probably because this sample was more hydrated, which might be related to its higher degree of ramification [16,17].

### 2.3. FT-IR Spectroscopy Studies

**Figure 3:**
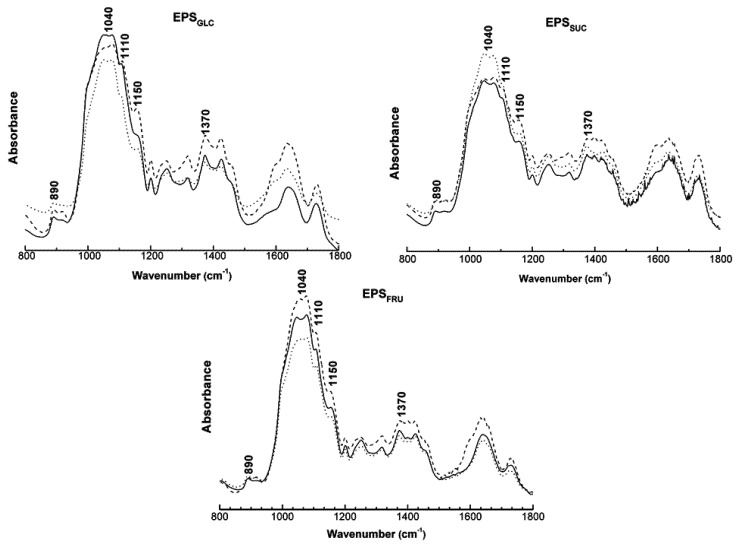
FT-IR Spectra for botryosphaerans EPS_GLC_, EPS_SUC_ and EPS_FRU_ before analysis (---), after TG (^___^) and after DSC (^….^), showing the characteristic bands of glucosidic linkages.

FT-IR was used to compare the results of analysis of the original EPS samples with those obtained after the samples were heated; in other words, before and after each thermal treatment. The spectra presented in Figure 3 showed similar results for all three of the botryosphaeran samples. The shoulder bands formed in the region between 1,500 and 900 cm^−1^ demonstrated there was no significant change (or alteration) of linkages corresponding to these bands. The bands at 1,040, 1,110 and 1,150 cm^−1^ corresponded to glucosidic linkages of the (1→3) type. The bands at 890 and 1,370 cm^−1^ corresponded to glucosidic linkages with a β-configuration. The characteristic β-glucan (1→3) linkages remained unchanged after each thermal treatment. The small variations that occurred in the spectra were not significant and were probably associated with the mild mass loss (removal of hydration water) and the endothermic transition found in the TG and DSC analyses, respectively.

### 2.4. Rheological Analysis

Rheological studies conducted on the different EPS produced by *B. rhodina* MAMB-05 demonstrated the pseudoplasticity of these biopolymers. Ding. *et al.* [6] studied the EPS produced by the bacterium *Erwinia chrysanthemi* using capillary viscometry to qualitatively compare EPS pseudoplasticity and rotational rheometry (cone-and-plate) to obtain η_0_ values using the Cross equation. Morris *et al. * [20] used the Cross Equation to extrapolate shear rate at zero, and found η_0_, for an EPS from the cyanobacterium *Aphanothece halophytica* GR02, and compared this with xanthan derived from *Xanthomonas campestris*.

**Figure 4:**
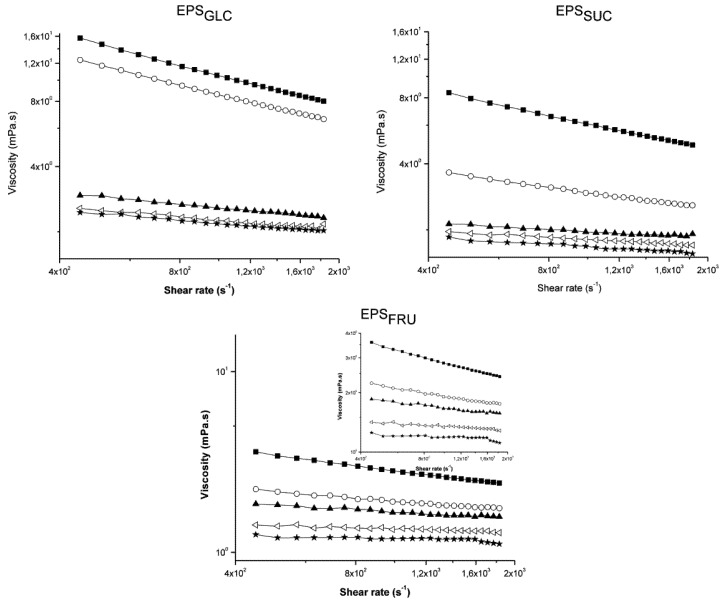
The apparent viscosity (η) *versus* shear rate (γ) in log scale for botryosphaerans at different concentrations: 1.0 g/L (-★-), 2.0 g/L (-▭-), 3.0 g/L(-▲-), 4.0 g/L (-○-) and 5.0 g/L (-■-). a) EPS_GLC_; b) EPS_SUC_; c) EPS_FRU_. The inset EPS_FRU_ graph is shown on a different scale to demonstrate “shear thinning” of EPS_FRU_.

Figure 4 shows a plot of the apparent viscosity (η) *versus* shear rate (γ) of the three botryosphaeran samples. At concentrations less than 3 g/L for EPS_GLC_ and EPS_SUC_, and less than 4 g/L for EPS_FRU_, the EPS apparent viscosities did not show any significant variations, just a slight decrease with a rise in γ, thus demonstrating an apparent Newtonian fluid behaviour. However, at concentrations of 4 g/L and 5 g/L for EPS_GLC_ and EPS_SUC_, and 5 g/L for EPS_FRU_, the viscosity of the EPS solutions declined (exponentially) with an increase in shear rate. This behaviour is consistent with pseudoplasticity, and was non-thixotropic because increases and decreases in the shear rate produced the same curve for each EPS concentration (data not shown).

The Power Law Equation [(1), Section 3.6] is one of the most widespread models in the literature for determining apparent viscosity data, and most of the calculation procedures related to non-Newtonian fluids are based on this equation [14,21]. Table 1 shows the k and ***a*** values for each EPS at each of the concentrations examined. 

**Table 1:** Values for consistency index or relaxation time (k) and Power Law index (*a*) for each concentration of the different botryosphaerans. These values confirmed the information presented in Figure 4.

Concentration (g/L)	EPS_GLC_		EPS_SUC_		EPS_FRU_	
	k *	*a*	k *	*a*	k *	*a*
1	0.005	0.863	0.002	0.891	0.002	0.966
2	0.006	0.860	0.003	0.900	0.002	0.960
3	0.008	0.833	0.003	0.921	0.003	0.891
4	0.180	0.560	0.015	0.736	0.006	0.834
5	0.265	0.533	0.084	0.621	0.018	0.730

* The unit of consistency index is mPa·s.

As all of the ***a*** values were found to be less than 1, this finding constitutes evidence of pseudoplasticity for each of the botryosphaerans. The ***a*** values at the three lowest concentrations of EPS examined were also close to 1, corroborating what was demonstrated in Figure 4. In other words, the EPS solutions tended to become Newtonian at low concentrations, in dilute regimes, as the ***a*** values approached 1. These viscometric properties were similar to those reported for xanthan and the EPS produced by several *E. chrysanthemi* species [22,23]. 

When tension is applied to a fluid constituted of macromolecules, the polymer chains adapt so that they become oriented in the direction of the flow. If the shear stress increases, the conformation of the biopolymer chains reaches maximum orientation. At this point, viscosity reaches a minimum and does not appear to be influenced by any further increases in shear stress [24]. Ding *et al. * [22] found similar rheological characteristics in a study with an EPS from *E. chrysanthemi*. 

In comparing the data obtained for each botryosphaeran (Figure 4), the apparent viscosity of EPS_GLC_ presented higher values than EPS_SUC_, which, in turn, was higher than EPS_FRU_. If η is large at low concentrations, the macromolecule must have a high axial ratio and, conversely, if η is small at high concentrations, the molecule must be somewhat compact [25]. The high η values of EPS_GLC_ suggested that its molecular axial ratio was larger than that of EPS_FRU_, which possessed a more compact axial ratio with more ramifications. The higher and lower k values (Table 1) also confirmed this conclusion, thus demonstrating that EPS_GLC_ had the highest viscosity and consistency in relation to the botryosphaerans produced on fructose and sucrose.

If η decreases significantly with increasing shear rate, the axial ratio must be high, because it is easier for a long and thin molecule to become oriented during flow than one that is short and thick [25]. Therefore, the more branched the molecule, the shorter and thicker it will be, and consequently, the more difficult its orientation during flow becomes. Less shear-thinning will therefore occur, suggesting that the degree of ramification of botryosphaeran produced on fructose as carbon source was higher than that produced when the fungus was grown on glucose. This finding was substantiated through the characterization of the chemical structure of these botryosphaerans [16,17].

The Cross Equation [(2), Section 3.6], a non-Newtonian mathematical model, has proved to give an excellent fit to the experimental flow curves of several polysaccharide solutions [26,27]. In these cases, the k constant was also related to the molecule’s structural relaxation time, which corresponds to the time it takes to break (during shearing) the entanglements formed by non-bonded interactions between the EPS molecular chains. The more concentrated the solution, the more intermolecular associations that EPS forms. Consequently, the freedom of movement of individual chains becomes progressively restricted, resulting in increases in the time required to form new entanglements to replace those disrupted by an externally imposed shear [7].

Pseudoplastic behaviour can be explained by the disruption of the structure formed by long polymer chains with an increase in the shear speed. These chains tend to align themselves in parallel to the normal orientation of the chains, decreasing the resistance to flow [14]. The k values for the three botryosphaerans are summarized in Table 1, from which it was concluded that the higher viscosity, the higher will be the molecular structural relaxation time.

The chemical structures of the botryosphaerans produced by *Botryosphaeria rhodina *MAMB-05, when grown on glucose, sucrose and fructose, have been determined [16,17]. According to the results of methylation and Smith degradation, EPS_FRU_ was shown to have the highest degree of ramification (see data presented in Table 2). The degree of ramification of EPS_GLC_ and EPS_SUC_ were very similar. Nevertheless, they were distinctly different in the type and extent of the branched substituents. In addition, gel permeation chromatography determined that EPS_GLC_ possessed a higher Mwthan the EPS produced on the other two carbon sources, with the EPS_FRU_ having the lowestMw.

**Table 2:** The degree of branching, proportion of glucose and gentiobiose branch units, elution volume, and η_0_ of the three botryosphaerans.

Samples	Extent of branching (%) *	Proportion of branches (%) *		Elution volume (mL) **	η_0_ of EPS solution (mPa.s) at substrate concentration of (g/L)	
		Glucose	Gentiobiose		1	5
EPS_GLC_	22	6	16	20.8	3.27	120.52
EPS_SUC_	21	12	9	21.3	2.08	33.24
EPS_FRU_	31	16	15	22.4	1.34	6.96

* From [17]; ** gel permeation chromatography on Sepharose CL-6B

Thus, a plausible explanation for the viscosity differences found between EPS_GLC_ and EPS_SUC_ can be influenced by the type, and degree, of ramification by the substituents on the interaction among the β-(1→3)-glucan chains. Added to this, the highest molecular weight of botryosphaeran was that produced on glucose (EPS_GLC_) according to gel filtration, and this also determined the highest viscosity.

### 2.5. Capillary Viscometry

Using the Martin Equation [(3), Section 3.6] and extrapolating the viscosity values of each botryosphaeran to zero, resulted in the logarithmic values of the intrinsic viscosity, which were in agreement with the sequence of higher and lower viscosity values found by rotational rheometry.

An empirical relationship exists between the weight-averaged molar mass (Mw) and the intrinsic viscosity [η], and is directly proportional for same-pair polymer solvent systems at the same temperature [24]. The intrinsic viscosity for EPS_GLC_ (54.95 dL/g) was much higher than that of EPS_FRU_ (16.98 dL/g). From this information we concluded that the Mw of EPS_GLC_ was highest, and that of EPS_FRU_ the lowest. EPS_SUC_ possessed a lower Mw than EPS_GLC_, but not greatly different, and this explains why its [η] value (41.69 dL/g) was close. These intrinsic viscosity values may be compared to literature values of other polysaccharides. For example, the EPS from *Propionibacterium acidi-propiocini* had an intrinsic viscosity of 22 dL/g that corresponded to a Mw of the order of 10^6^ g/mol [7]. An intrinsic viscosity value of 45 dL/g was found for an EPS from red microalga [28] with an expected Mw of ~6 × 10^6^ g/mol, while the intrinsic viscosity of oat β-glucans was lower and varied between 0.67 and 3.38 dL/g with the Mw ranging from 35 to 250 × 10^3^ g/mol [11]. These literature findings allowed us to infer that the botryosphaerans have a high Mw, and this might explain why they become extremely viscous in aqueous solutions at low concentrations. The Mw reported for a polysaccharide from another *B. rhodina* strain (viz., DABAC-P82) was of the order of 4.875 × 10^6^ [3], thus indicating the high Mw of the polysaccharides produced by *B. rhodina*.

The conformation of polysaccharides in solutions, especially in aqueous solutions, can be investigated according to the theory of dilute polymer solutions. The intrinsic viscosity [η] is a characteristic property of polysaccharide solution [29,30]. Intrinsic viscosity also provides a convenient measure of the hydrodynamic volume of individual polymers coils, and when this is multiplied by concentration gives an index of total degree of “space-occupancy”, the reduced concentration (c[η]). For the three botryosphaerans the hydrodynamic volume of EPS_FRU_ showed lower values, while EPS_GLC_ and EPS_SUC_ had fairly similar values over the ranges studied (data not shown). In aqueous solution, linear chains expand (“space-occupancy”) much more than branched chains because the linkages among the chains limit their expansion coefficients [24]. From this information it was concluded that EPS_FRU_ was the most branched, as it possessed the lowest hydrodynamic volume among the botryosphaerans produced on the three carbon sources studied.

## 3. Experimental

### 3.1. Production of Botryosphaeran

Botryosphaerans (EPS) was produced by cultivating the fungus *Botryosphaeria rhodina* MAMB-05 on minimal salts medium containing either glucose (EPS_GLC_), fructose (EPS_FRU_) or sucrose (EPS_SUC_) as the sole carbon source, and were recovered from the cell-free culture fluid by precipitation with 4 volumes of ethanol as described previously [16,17].

### 3.2. Differential Scanning Calorimetry (DSC)

DSC measurements were carried out with a Netzsch 204 Phoenix Thermal Analyzer (Netzsch -Gerätebau GmbH, Selb, Germany). All measurements were conducted in duplicate using hermetically sealed 25 μL volume aluminium pans, containing 4 mg of each dried EPS sample (EPS_GLC_, EPS_SUC_ and EPS_FRU_). An empty aluminium pan was used as reference. DSC analyses were performed under nitrogen at a flow rate of 20 mL/min. Samples were heated at a rate of 5 °C/min from −50 °C to 150 °C, and were then kept isothermal for 30 min. Derivative curves of DSC (DDSC) were recorded, and the thermograms analysed using the Proteus Software for Thermal Analysis (Netzsch) for Microsoft Windows 95/98 NT 4.0/2000.

### 3.3. Thermogravimetric (TG) Analysis

TG measurements were carried out with a Netzsch 209 Iris Thermal Analyzer (Netzsch). Measurements were performed in duplicate using 40-μL volume open aluminium pans containing 5 mg of each dried sample (EPS_GLC_, EPS_SUC_ and EPS_FRU_). TG analyses were conducted under nitrogen at a flow rate of 15 mL/min. Heating was performed in the range of 30–150 °C with a temperature rate increase of 5 °C/min, and then maintained at 150 °C for 30 min. Derivative curves of TG (DTG) were recorded and analysed using Proteus Software for Thermal Analysis.

### 3.4. Fourier-Transform Infra-red Spectroscopy (FT-IR)

FT-IR spectroscopy was carried out using a Bruker Vector 22 Model FT-IR Spectrometer (Bruker Optik GmbH, Ettlingen, Germany) on 4–5 mg of each freeze-dried botryosphaeran sample in 250 mg of KBr. Scans were performed within the 1,800–800 cm^−1^ range at a resolution of 2 cm^−1^. Spectra were taken before and after each thermal analysis, and OPUS/IR NT4.0 Spectroscopic Software was used to control the spectrometer and for the acquired and manipulated spectra. 

### 3.5. Gel permeation Chromatography

Solutions of each EPS (1 g/L) were centrifuged at 3000 × *g*/10 min, and 1 mL aliquots applied to a column of Sepharose CL-6B (23.5 cm × 1.6 cm). The column was then eluted with distilled water at a flow rate of 0.52 mL/min, and 2.5 mL fractions collected. The void volume (17.9 mL) of the column was determined with Blue Dextran [16,17].

### 3.6. Rheology

Rotational rheometer measurements were performed in triplicate on aqueous solutions of botryosphaeran at concentrations of: 1, 2, 3, 4 and 5 g/L at 25 °C using a Brookfield rotational rheometer (RV DV-III model, Brookfield Engineering Laboratories, Stoughton, MA, USA) equipped with a cone-plate attachment (spindle CP 40) operating at shear rates within the range of 450–1830 s^−1^ and at speeds from 60–244 rpm. Rheological data was acquired via a personal computer using the Rheocalc V2.3 software (Brookfield Engineering Laboratories). 

The Power Law equation used to analyse the rheological behaviour of the EPS is described by:

τ = kγ *^a^* (1)

where τ is the shear stress, k is the consistency index (apparent viscosity at a shear rate of 1 s^−1^), γ is the shear rate, and ***a*** represents a constant that characterizes a material. If ***a*** = 1, the fluid is Newtonian; if ***a*** < 1, pseudoplastic; and if ***a*** > 1, dilatant. In relation to k, the higher its value, the more consistent (or viscous) is the fluid.

The Cross equation was used to obtain appropriate values for zero-shear rate viscosity (η_0_) by extrapolation of the experimental data. The Cross equation is given by:

η = η_∞_ + [(η_0_ − η_∞_) / (1 + kγ*^a^*)] (2)

where η_∞_ is the limiting viscosity estimated at infinite shear rate. The k, γ and ***a*** symbols are the same as aforementioned.

The Martin equation:

log(η_sp_/c) = log[η] + K_M_ [η]c (3)

where η_sp_ is the specific viscosity, [η] is the intrinsic viscosity, and K_M_ is a dimensionless parameter that depends upon the given polymer-solvent pair, was used to determine the intrinsic viscosity. An Ubbelodhe Cannon E-534 Viscometer (Cannon Instrument Co, State College, PA, USA) was used for capillary viscometric analysis. Each concentrated exopolysaccharide solution (10 g/L) was prepared by dispersing the dried hydrocolloids in water at 25 °C under constant stirring and diluting to concentrations of 0.1, 0.5, 0.75, 1.0, 1.5, 2.0 and 2.5 g/L. The analyses were performed in triplicate, at a temperature of 25 °C, in 10 min. intervals.

## 4. Conclusions

Under the conditions examined, the botryosphaerans produced by *B. rhodina* MAMB-05 grown on glucose and sucrose showed similar thermal behaviour, inasmuch as both EPSs presented slight endothermic variation and mass loss on thermal analysis. Small variations in the respective FT-IR spectra after heating were also observed, and most probably were a consequence of the removal of water of hydration. As none of the botryosphaerans samples presented exothermic peaks, large mass loss and/or significant spectral alterations after thermal analysis, this suggested that the EPS_GLC_, EPS_SUC_ and EPS_FRU_ possessed good thermal stability in the temperature range generally used to sterilise biomaterials. In addition, the species with the highest molecular mass, EPS_GLC_ (determined by gel filtration), was influenced by the effect of the type and degree of ramification on the strengths of the intermolecular interactions among the exopolysaccharide chains, and can be a plausible explanation for the viscosity differences found among the three botryosphaerans. The higher k values of EPS_GLC_ also suggested a higher structural relaxation time than of EPS_SUC_ and EPS_FRU_.
